# The role of metacognitive beliefs in generalised anxiety disorder in men who have sex with men living with HIV in Nigeria

**DOI:** 10.1177/13591053251314989

**Published:** 2025-02-19

**Authors:** Robin Bailey, Ekwora Chinonso Oba, Rosie Allen

**Affiliations:** School of Psychology, Deane Road, University of Bolton, Bolton, UK

**Keywords:** generalised anxiety disorder, HIV, metacognitive beliefs, Nigeria, stigma

## Abstract

Men who have sex with men (MSM) living with HIV tend to experience a range of mental health issues, in particular generalised anxiety disorder (GAD), often caused and maintained by psychosocial variables including HIV stigma, discrimination, self-esteem issues, substance abuse and loneliness. This is particularly problematic in countries like Nigeria where same sex activity is illegal and can result in up to 14 years imprisonment. An important psychological variable that may contribute to the experience of GAD are metacognitive beliefs. Participants (*N* = 311) completed measures to examine the relationship between these variables. Results indicated that metacognition was associated with, and significantly predicted, GAD in this population. Moderation analysis showed that the effect of HIV stigma on GAD was explained by the proposed interaction with metacognition. Findings suggest that metacognition may be an important variable in explaining GAD symptoms in MSM living with HIV in Nigeria.

## Introduction

Contracting the Human Immunodeficiency Virus (HIV) is now regarded as a chronic illness that can be managed, rather than a death sentence (Mendonca et al., 2023). This is due to the effective use of anti-retroviral therapies in reducing the mortality rate of HIV – a disease which was once acute and fatal (Sematlane et al., 2021). As a chronic disease, HIV presents additional challenges outside the physical effects, for individuals living with this condition.

As much progress has been made internationally to reduce the stigma associated with being homosexual and having HIV, Nigeria, a country with rich cultural diversity and strong religious morality, remains a stagnant environment for such change. For those living with HIV and who are gay, this challenging environment presents a range of social, cultural, emotional and psychological challenges (Awofala and Ogundele, 2018; Sekoni et al., 2022).

In Nigeria, men who have sex with men (MSM) are estimated to total 1% of the general population, with 20% of new HIV infections being recorded in MSM (Eluwa et al., 2019). Also, same sex activity is illegal in Nigeria as legislated by the ‘Same-Sex Marriage Prohibition Act, 2013’ (Oguntola-Laguda and van Klinken, 2016). This can result in up to 14 years imprisonment for anyone who ‘[enters] into a same-sex marriage contract or civil union’, and a 10-year prison sentence for those who ‘witness, abet, and aid the solemnization of a same-sex marriage or union’ (Igundunasse et al., 2019). The effects of homosexuality being criminalised has resulted in excessive harassment, human rights violations and physical violence against homosexual individuals (Strömdahl et al., 2019). The prevalence of suicidal ideation among people living with HIV in Nigeria is 13.6%, with this figure rising to 30.5% among sexual minorities (Oginni et al., 2019; Ogundipe et al., 2015; Lee et al., 2011). In comparison, the rates of suicidal ideation are 10% in the United States, 26% among sexual minorities in the U.S. and 15% in Thailand.

In general, the impact of chronic illnesses on psychological wellbeing is well documented (Bahall and Bailey, 2022; Hwu, 1995; Xiao et al., 2022), however, for MSM living with HIV in Nigeria these effects can be amplified due to the stigma, discrimination and the illegal status of homosexuality (Folayan et al., 2022).

One mental health condition that has been implicated in people living with HIV is Generalised Anxiety Disorder (GAD). GAD is a type of anxiety disorder characterised by persistent and excessive worry that is hard to bring under control (American Psychiatric Association Division of Research, 2013). In a USA based study, prevalence rates for individuals with HIV who experience symptoms of GAD is 19%, with symptoms consistent with a diagnosis of GAD being present in one in five individuals (Beer, 2019). A study by Heywood and Lyons (2016) reports high rates of anxiety among gay men in Australia living with HIV, with a significant number of them not receiving treatment or diagnosis. In a similar study, a high incidence of anxiety was reported among MSM with HIV in the United Kingdom and Ireland (Murphy et al., 2018). In a Nigerian based study, prevalence rates for GAD were 25.6%, indicating rates higher than those reported in other prevalence studies (Aika and Odili, 2019). Many negative effects have been documented in the relationship between GAD and HIV, such as poor adherence to Antiretroviral therapy and poor attendance at HIV-care appointments (Mannes et al., 2019; Tucker, 2003), increased risk for HIV transmission and increased hospitalisations (Beer, 2019).

Focusing on GAD is particularly relevant because of its significant impact on both physical health outcomes and psychological well-being in this patient group. By exploring GAD specifically, it may help us understand which variables contribute most to the disorder, and to then develop more effective interventions that not only address anxiety symptoms but also improve HIV-related health outcomes.

Stigma has been considered a key variable in predicting GAD in MSM living with HIV. Findings from the 2013 ‘Nigeria Demographic Health Survey’ showed that about half of Nigerians living with HIV/AIDS experience stigma (Dahlui et al., 2015), while a more recent study indicated this figure to be 73.7% (Ahmed et al., 2022). The stigma associated with both homosexuality and HIV can exacerbate the symptoms of GAD among this group (Rodriguez-Hart et al., 2018) more so than heterosexual individuals (Lee et al., 2022; Rodriguez-Seijas et al., 2019). Individuals of a sexual minority who live in a country that openly discriminates and legislates against homosexuality often feel forced to live discreetly as an act of self-protection to avoid persecution (Pachankis and Bränström, 2018). The impact of such concealment is linked and strongly correlated with mental health problems such as GAD (Pachankis, 2020). Self-directed homophobia, or internalised stigma to one’s own sexual orientation, can significantly contribute to further GAD related symptomatology (Wen and Zheng, 2019), which can often be the case for sexual minorities living in a country that criminalises homosexuality (Hersh, 2015). In one study, multiple linear regressions revealed that anxiety was positively predicted by internalised stigma and enacted stigma, with internalised stigma being the strongest and most significant predictor (Murphy et al., 2018).

Other psycho-social challenges faced by MSM living with HIV which contribute to poor mental health (Mo et al., 2020; Murphy et al., 2018; Wang et al., 2019) include loneliness, self-esteem and substance abuse. With high levels of homophobia, HIV stigma and mental health discrimination, MSM with HIV can often experience the negative effects of loneliness (Austin-Keiller et al., 2023) and social isolation (Hubach et al., 2015). Constantly pre-empting discrimination and rejection, this group tend to avoid social interactions or starting relationships (Fish and Weis, 2019). On top of loneliness, MSM living with HIV will also have specific issues related to self-identity and self-esteem, with one study highlighting this being particularly problematic in Nigeria (Folayan et al., 2022). The dual task of managing both HIV and the internal complexities of sexual identity can lead to an eroding of an individual’s self-esteem (Handayani and Susanti, 2019). With studies finding a negative correlation between self-esteem and anxiety in gay and bisexual men living with HIV (Agyemang et al., 2020; Batista and Pereira, 2020). The societal belief that HIV is a punishment and the fact that being gay is punishable by law heightens internalised homophobia and diminishes self-worth (Tan, 2019).

Individuals living with HIV often have comorbid substance abuse issues that can have an impact upon their mental health (O’Cleirigh et al., 2015), and particularly their symptoms of GAD, as supported by the following studies. Alcohol abuse has been observed in MSM living with HIV and has shown to be problematic, resulting in issues with health, disease management and medication compliance (Arasteh et al., 2008; Chander et al., 2006; Galvan et al., 2002; Monroe et al., 2016). In an USA based study by Mannes et al. (2021) investigating GAD as a risk factor for substance use in adults living with HIV, the authors found that those with moderate to severe symptoms of GAD had significantly higher odds of engaging in substance use compared to those with none or mild symptoms. Specifically, individuals with moderate/severe GAD had 1.7 times greater odds of smoking cigarettes (AOR = 1.70, 95% CI = 1.18–2.45, *p* = 0.004), 1.5 times higher odds of hazardous alcohol consumption (AOR = 1.50, 95% CI = 1.04–2.16, *p* = 0.028) and 1.75 times greater odds of using crack or cocaine (AOR = 1.75, 95% CI = 1.13–2.69, *p* = 0.011). These findings suggest that as symptoms of GAD increase, so does the likelihood of substance abuse, indicating a strong relationship between anxiety severity and substance use behaviours in this population. In an Indian based study relationships between GAD, HIV and substance abuse have been considered bi-directional, in that HIV plus substance abuse increases risk for GAD and GAD increases risk for HIV plus substance abuse (Singh et al., 2020).

Outside of substance abuse, societal factors such as stigma and discrimination, and emotional factors such as loneliness and self-esteem, individual psychological mechanisms may well be important in the maintenance of high levels of GAD within this group. One such mechanism may be the individuals’ metacognitive beliefs.

Metacognitive beliefs encompass one’s thoughts and perceptions about cognitive processes and have been shown to be one of the most important psychological mechanisms in GAD (Sun et al., 2017). These beliefs influence how individuals evaluate their thoughts, particularly those related to worry and perceived threats, and thus can either promote adaptive coping or contribute to emotional dysregulation (Hoffart et al., 2018). The concept of metacognition is crucial because it goes beyond traditional cognitive theories seen in CBT that focus on the content of thoughts, by emphasising the process of thinking about thinking, making it a more comprehensive framework for understanding emotional disorders (Wells, 2019). Metacognitive beliefs have been show to explain GAD more than traditional CBT based cognitions (Khawaja and McMahon, 2011).

According to the metacognitive model of GAD (Nordahl et al., 2023; Wells, 2005), individuals hold positive metacognitive beliefs about the usefulness of worry that is, ‘Worry helps me cope’ (Capobianco et al., 2020; Wells, 2011). They also hold negative metacognitive beliefs that involve excessive worry about the uncontrollability of thoughts, the dangerousness of worry and negative beliefs about worry itself (Clauss et al., 2020). Triggers that activate positive beliefs about the necessity of worrying often include intrusive negative thoughts, such as imagining being discriminated against, or external factors like someone shouting verbal abuse. These are commonly referred to as type 1 worry (Wells, 2005). However, according to the model it is the development of negative beliefs about worrying that contributes centrally to the transition to GAD. These beliefs lead to negative appraisal of worry itself, a term described as ‘meta-worry’ or ‘type 2 worry’ (Wells, 2011), such as ‘I am going mad with worry’. Individuals will often engage in maladaptive behaviours as a means of trying to control the worry for example, avoidance of situations and people and substance abuse.

One study has previously looked at the role of metacognitions in relation to HIV stigma and depression and anxiety in MSM with HIV (Strodl et al., 2015). Using an Australian sample of 106 gay, bisexual and other MSM living with HIV, they found negative metacognitive beliefs mediated the association between internalised HIV stigma and anxiety symptoms. Negative metacognitive beliefs appear to be a risk factor in understanding the psychological vulnerability in response to HIV stigma within some MSM living with HIV. In this study however, they only controlled for demographic variables, rather than other established variables that may lead to anxiety in this population. Also, the authors concluded that detecting significant results may have been restricted due to the use of a smaller sample size (*N* = 106), and in-depth analyses with larger samples are recommended within future studies. Also, as the sample was predominantly from a country where homosexuality is legal, levels of stigma may be very different from those experienced in a country like Nigeria, where homosexuality can result in a 14-year prison sentence. So, whether metacognition remains a predictor of anxiety in a country and culture where anti-gay law is associated with increased stigma and discrimination, needs exploring.

Compared to other theoretical approaches, for example CBT, metacognitive theory is more useful in explaining why individuals with GAD often continue to experience persistent worry despite being aware that worrying is problematic (Thielsch et al., 2015). It shifts the focus from challenging the content of their thoughts to changing the relationship with worry itself, making it particularly relevant for populations facing chronic stressors, such as MSM living with HIV. Understanding how metacognitive beliefs operate in these populations could lead to more targeted and effective psychological interventions, potentially improving treatment outcomes for GAD within this population.

To date there is still a gap in knowledge regarding the relationship between metacognitive beliefs and generalised anxiety in some sub populations like MSM with HIV in Nigeria. Examining these beliefs within this specific demographic can offer insights into the psychological processes that may contribute to GAD, particularly when controlling for the other variables associated with psychosocial distress that is, stigma, discrimination, loneliness, self-esteem and substance abuse.

As such it is hypothesised that: (a) Metacognitive beliefs will be positively correlated with generalised anxiety disorder symptoms in this population. (b) Positive beliefs about worry and negative beliefs about uncontrollability and danger will significantly predict symptoms of GAD over and above socio-emotional factors such as stigma, discrimination, substance abuse, loneliness and self-esteem. (c) The relationship between type 1 worry that is, HIV stigma and generalised anxiety, will be positively moderated by negative beliefs about uncontrollability and danger.

## Method

### Participants and procedure

Ethics approval for this study was granted by the University Ethics Committee. The study comprised 311 men who are clients of a non-governmental sexual and reproductive health clinic located in Lagos, specialising in providing free healthcare services to individuals with HIV/AIDS. Confirmation of participants’ HIV serostatus was based on records available at the facility. The participants of this study were eligible if they were male, residents of Lagos State, identified as having had sex with another male within the past year, living with HIV and willing to give consent to the study. Recruitment occurred during their scheduled appointments at the centre. The study details were initially communicated to the participants by the community mobilisation officers representing the organisation. Following this introduction, participants engaged in the informed consent procedures, which were comprehensively explained to them. To ensure clarity and transparency, participants were provided with detailed information about the study’s objectives, procedures and any associated risks or benefits. This information was also provided via a Google document link, allowing participants to access the study details and consent form online. Through this online link, participants provided written informed consent and were able to fill out the questionnaire and the consent form.

### Measures

The measures used in this study were selected for their strong psychometric properties and specific to the associated psychosocial variables of MSM living with HIV. The *Metacognitions Questionnaire-30* (MCQ-30; Wells and Cartwright-Hatton, 2004) assesses metacognitive beliefs that are key to understanding GAD, with well-established reliability across diverse populations (Nordahl et al., 2022). The *Generalised Anxiety Disorder-7* (GAD-7; Spitzer et al., 2006) was chosen for its efficiency and validity in measuring anxiety severity, making it ideal for screening GAD in clinical and general populations, including those with HIV (Nyongesa et al., 2020). To assess HIV-related stigma, the *Berger HIV Stigma Scale* (Berger et al., 2001) captures the multidimensional nature of stigma experienced by HIV-positive individual and is culturally applicable (Oke et al., 2019), while the *Discrimination and Stigma Scale version 12* (DISC-12; Thornicroft et al., 2009) provides insight into experiences of discrimination across various life domains, critical for understanding the psychosocial stressors in this population. The *De Jong Gierveld Loneliness Scale* (DJGLS; De Jong Gierveld and Van Tilburg, 1999) was included to assess loneliness, a significant factor in MSM’s mental health, particularly in HIV groups (Gordijn and Boven, 2009). The *Rosenberg Self-Esteem Scale* (Rosenberg, 1965) was chosen to measure self-esteem, and used frequently in this client group (Adimora et al., 2019). Lastly, the *Drug Abuse Screening Test-20* (DAST-20; Skinner and Goldberg, 1986) was used to assess substance abuse, which often co-occurs with anxiety and serves as a maladaptive coping mechanism for HIV-related stress. Together, these validated measures provide a comprehensive assessment of the psychosocial and cognitive factors contributing to GAD in this population.

#### Metacognitions questionnaire-30 (MCQ-30)

The MCQ-30 (Wells and Cartwright-Hatton, 2004) is a self-report measure of metacognitive beliefs comprising 30 items with four-choices ranging from ‘I don’t agree’ = 1 to ‘I completely agree’ = 4. The measure consists of five subscales: ‘Positive beliefs about worry’; ‘Negative beliefs about worry’; ‘Cognitive confidence’; ‘Need to control thoughts’; and ‘Cognitive self-consciousness’ which have been confirmed across a variety of countries and cultures (Nordahl et al., 2022). The MCQ-30 has acceptable-to-excellent internal consistency (subscale αs from 0.72 to 0.93), consistent factor structure, and convergent validity with other measures of maladaptive metacognition (Huntley et al., 2022).

#### Drug Screening Questionnaire-20 (DAST-20)

The Drug Abuse Screening Test -20 (DAST-20) is a self-report measure used to assess the extent of the problems related to drug misuse such as such as employment, medical conditions and family and social interactions (Cusimano et al., 2020; Skinner and Goldberg, 1986). The test uses a binary response, requiring either a ‘Yes’ or a ‘No’ response to each question. Scores of 6 or above indicate a drug use disorder. The DAST-20 includes five sub-factors (dependence, social difficulties, medical problems, poly-drug use previous treatment; Yamada et al., 2021). The DAST-20 has good internal consistency (range 0.71 to 0.99), and convergent validity (−0.13 to 0.75) making it a reliable screening tool (Johnson, 2021).

#### The Discrimination and Stigma Scale version 12 (DISC-12)

The Discrimination and Stigma Scale version 12 (DISC-12; Thornicroft et al., 2009) is a 34-item standardised measure for assessing discrimination. The DISC-12 questions participants about their encounters with discrimination in several aspects of their lives, such as jobs, mental health treatment, relationships with friends, neighbours and relatives. The measure has an overall global scale and four subscales: Unfair treatment; Stopping self; Overcoming stigma; and Positive treatment. The DISC-12 responses are scored on a 4-point Likert scale: not at all = 0, a little = 1, moderately = 2 and a lot = 3. In this study only the quantitative element was scored, with higher scores indicating higher levels of discrimination. The measure has established psychometric properties such as high internal consistency (α = 0.78) and test-retest reliability (range: 0.56–0.89) (Ebuenyi et al., 2019).

#### Berger HIV stigma scale

The Berger HIV stigma scale (Berger et al., 2001) is a 40 item self-report measure assessing levels of perceived HIV stigma. The Berger scale measures perceived stigma using a 4-point Likert scale (strongly disagree = 1, disagree = 2, agree = 3, strongly agree = 4). The scale consists of four subscales: personalised stigma; disclosure concerns; negative self-image; and concern with public attitudes. In a recent meta-analysis, the reported internal consistency of the HIV stigma scale ranged from acceptable to excellent (Cronbach’s alpha ≥0.70) and was considered a reliable and valid measure of HIV-related stigma (Wanjala et al., 2021).

#### GAD-7 anxiety scale

GAD-7 is a seven-item self-rating scale screening for and measuring anxiety, reflecting the symptom criteria for generalised anxiety disorder (GAD; Simoen et al., 2020; Spitzer et al., 2006). The GAD-7 is scored on a 4-point Likert scale (0–3) with total scores ranging from 0 to 21 with higher scores indicating greater anxiety severity. The Gad-7 has been shown to be a reliable and valid measure of GAD symptoms in psychiatric (Rutter and Brown, 2017) and general population (Hinz et al., 2017) samples. The scale has been found to have good internal reliability (Cronbach’s alpha = 0.90; Spitzer et al., 2006).

#### Rosenberg Self-Esteem Scale

Rosenberg’s Self-Esteem Scale (Rosenberg, 1965) is a 10-item scale that assesses overall self-worth by assessing one’s feelings of self-esteem, self-competence and self-acceptance (Hutz and Zanon, 2011). Five of its items are worded positively, whereas the remaining five are worded negatively (Rosenberg, 1965). This measure’s items are graded on a 4-point Likert scale ranging from strongly agree to strongly disagree. The study has been used in multiple cross-cultural contexts (Schmitt and Allik, 2005).

#### De Jong Gierveld Lonliness Scale

The De Jong Gierveld Loneliness Scale (DJGLS; De Jong Gierveld and Van Tilburg, 1999) is a tool used to assess the many aspects of loneliness experienced by individuals. It encompasses the emotional and social components of loneliness in two dimensions. It has five positive and six negative statements, each on a 5-point Likert scale. DJGLS results are divided into three categories: emotional loneliness (0–6 points), social loneliness (0–5 points) and overall loneliness (0–11 points), which is the sum of the previous two. A higher number of points in each area implies that the individual feels lonelier. Internal reliability has already been determined to be satisfactory for both the global scale and both subscales (all Cronbach’s >0.80; Thompson and Pollet, 2024). Internal reliability was found to be good for the subscales of emotional loneliness (Cronbach’s = 0.89) and social loneliness (Cronbach’s = 0.88; Thompson and Pollet, 2024).

### Statistical analysis

Initially we ran correlations between our dependent variable, generalised anxiety and the independent variables HIV stigma, mental health discrimination, loneliness, self-esteem and five metacognitive variables (lack of cognitive confidence, positive beliefs about worry, cognitive self-consciousness, negative beliefs about uncontrollability and danger and need to control thoughts).

A hierarchal regression analysis was conducted to explore whether specific metacognitive beliefs explain variance in GAD beyond psycho-social factors.

Next, we tested metacognitive moderation using bootstrapping and the PROCESS tool designed by Hayes (2022). PROCESS is a computational macro used with SPSS for path analysis-based moderation. This tool enables a more modern and up to date procedure for moderation analysis (Hayes, 2009). PROCESS command model – 1 was used as this estimates a moderation model with a single moderator of the effect of X (HIV stigma) on Y (GAD) by M (negative beliefs about uncontrollability and danger).

## Results

### Inter-correlations

The data were screened to check for normality. A comprehensive examination was conducted on the frequency histograms, and findings from a Kolmogorov-Smirnov test revealed that the data were not normally distributed. As a result, Spearman’s Rho was employed as the correlational test.

Results (Table 1) showed significant positive correlations between all variables and generalised anxiety, in particular HIV stigma (*r* = 0.64, *p* < 0.001) and discrimination (*r* = 0.61, *p* < 0.001). All five metacognitive beliefs were significantly correlated with GAD with negative beliefs about uncontrollability and danger (*r* = 0.60, *p* < 0.001) and positive metacognitive beliefs (*r* = 0.56, *p* < 0.001) being the strongest. This is in line with previous research which identifies both positive and negative beliefs as being particularly problematic in GAD (Sun et al., 2017).

**Table 1.** Inter-correlations.

Correlation	IV		GAD	HIV	Disc	SAbuse	Lone	SelfE	LOCC	PBAW	CSC	NBUD	NTCT
Spearman’s rho	GAD	Correlation coefficient	1.000	0.644**	0.606**	0.540**	0.378**	0.173**	0.509**	0.558**	0.207**	0.596**	0.518**
		Sig. (2-tailed)	–	<0.001	<0.001	<0.001	<0.001	0.002	<0.001	<0.001	<0.001	<0.001	<0.001
		*N*	311	311	311	311	311	311	311	311	311	311	311
	HIV	Correlation coefficient	0.644**	1.000	0.724**	0.546**	0.366**	0.240**	0.668**	0.687**	0.293**	0.672**	0.645**
		Sig. (2-tailed)	<0.001	–	<0.001	<0.001	<0.001	<0.001	<0.001	<0.001	<0.001	<0.001	<0.001
		*N*	311	311	311	311	311	311	311	311	311	311	311
	Disc	Correlation coefficient	0.606**	0.724**	1.000	0.622**	0.378**	0.244**	0.584**	0.660**	0.138*	0.539**	0.485**
		Sig. (2-tailed)	<0.001	<0.001	–	<0.001	<0.001	<0.001	<0.001	<0.001	0.015	<0.001	<0.001
		*N*	311	311	311	311	311	311	311	311	311	311	311
	SAbuse	Correlation coefficient	0.540**	0.546**	0.622**	1.000	0.237**	0.327**	0.536**	0.545**	−0.039	0.466**	0.429**
		Sig. (2-tailed)	<0.001	<0.001	<0.001	–	<0.001	<0.001	<0.001	<0.001	0.492	<0.001	<0.001
		*N*	311	311	311	311	311	311	311	311	311	311	311
	Lone	Correlation coefficient	0.378**	0.366**	0.378**	0.237**	1.000	0.092	0.293**	0.324**	0.141*	0.316**	0.245**
		Sig. (2-tailed)	<0.001	<0.001	<0.001	<0.001	–	0.105	<0.001	<0.001	0.013	<0.001	<0.001
		*N*	311	311	311	311	311	311	311	311	311	311	311
	SelfE	Correlation coefficient	0.173**	0.240**	0.244**	0.327**	0.092	1.000	0.301**	0.188**	−0.251**	0.169**	0.167**
		Sig. (2-tailed)	0.002	<0.001	<0.001	<0.001	0.105	–	<0.001	<0.001	<0.001	0.003	0.003
		*N*	311	311	311	311	311	311	311	311	311	311	311
	LOCC	Correlation coefficient	0.509**	0.668**	0.584**	0.536**	0.293**	0.301**	1.000	0.757**	0.192**	0.706**	0.695**
		Sig. (2-tailed)	<0.001	<0.001	<0.001	<0.001	<0.001	<0.001	–	<0.001	<0.001	<0.001	<0.001
		*N*	311	311	311	311	311	311	311	311	311	311	311
	PBAW	Correlation coefficient	0.558**	0.687**	0.660**	0.545**	0.324**	0.188**	0.757**	1.000	0.282**	0.683**	0.618**
		Sig. (2-tailed)	<0.001	<0.001	<0.001	<0.001	<0.001	<0.001	<0.001	–	<0.001	<0.001	<0.001
		*N*	311	311	311	311	311	311	311	311	311	311	311
	CSC	Correlation coefficient	0.207**	0.293**	0.138*	−0.039	0.141*	−0.251**	0.192**	0.282**	1.000	0.340**	0.398**
		Sig. (2-tailed)	<0.001	<0.001	0.015	0.492	0.013	<0.001	<0.001	<0.001	–	<0.001	<0.001
		*N*	311	311	311	311	311	311	311	311	311	311	311
	NBUD	Correlation coefficient	0.596**	0.672**	0.539**	0.466**	0.316**	0.169**	0.706**	0.683**	0.340**	1.000	0.742**
		Sig. (2-tailed)	<0.001	<0.001	<0.001	<0.001	<0.001	0.003	<0.001	<0.001	<0.001	–	<0.001
		*N*	311	311	311	311	311	311	311	311	311	311	311
	NTCT	Correlation coefficient	0.518**	0.645**	0.485**	0.429**	0.245**	0.167**	0.695**	0.618**	0.398**	0.742**	1.000
		Sig. (2-tailed)	<0.001	<0.001	<0.001	<0.001	<0.001	0.003	<0.001	<0.001	<0.001	<0.001	–
		*N*	311	311	311	311	311	311	311	311	311	311	311

GAD: generalised anxiety; HIV: HIV stigma; Disc: discrimination; SAbuse: substance abuse; Lone: loneliness; SelfE: self esteem; LOCC: lack of cognitive confidence; PBAW: positive beliefs about worry; CSC: cognitive self consciousness; NBUD: negative beliefs about uncontrollability and danger; NTCT: need to control thoughts; IV: Independent Variable.

** Correlation is significant at the 0.01 level (2-tailed).

* Correlation is significant at the 0.05 level (2-tailed).

### Hierarchical regression analysis

To investigate the relative contribution of metacognitive beliefs to generalised anxiety in this population, a hierarchical regression analysis was run. To begin with we tested for multicollinearity by examining variance inflation factors (VIFs) and tolerance statistics. After entering the predictors all seemed acceptable, with tolerance values emerging as greater than the recommended 0.2 (range 0.33–0.87; Menard, 2000) and all VIF values were less than 10 (range 1.14–3.04; Myers, 1990).

With the GAD-7 as the dependent variable (Table 2), on step 1, the HIV-Stigma and mental health discrimination was force entered into the equation to control for societal variables and explained 44% of variance and was significant. On step 2, loneliness, self-esteem and substance abuse were force entered as a block to control for social/emotional variables and explained 4.7% of the variance. Finally, the five metacognitive subscales were entered into the equation. The block of metacognitive beliefs explained a further significant 3.1% of the variance in GAD (Table 2). Overall, the full model accounted for 51.8% of the variance in GAD (Multiple *R* = 0.72, *F*(10, 300) = 32.29, *p* < 0.001).

**Table 2.** Regression coefficients.

Model		Unstandardised coefficients		Standardised coefficients	*t*	Sig.
		*B*	Std. Error	β		
1	(Constant)	−3.779	1.092		−3.461	<0.001
	HIV	0.069	0.012	0.364	5.747	<0.001
	Disc	0.088	0.016	0.348	5.504	<0.001
2	(Constant)	−3.395	2.679		−1.267	0.206
	HIV	0.056	0.012	0.295	4.718	<0.001
	Disc	0.055	0.017	0.217	3.283	0.001
	SAbuse	0.340	0.081	0.225	4.184	<0.001
	Lone	0.122	0.036	0.149	3.375	<0.001
	SelfE	−0.076	0.105	−0.032	−0.731	0.465
3	(Constant)	−5.995	3.091		−1.939	0.053
	HIV	0.034	0.014	0.178	2.448	0.015
	Disc	0.058	0.017	0.229	3.459	<0.001
	SAbuse	0.316	0.084	0.209	3.780	<0.001
	Lone	0.099	0.036	0.122	2.762	0.006
	SelfE	−0.019	0.113	−0.008	−0.167	0.867
	LOCC	−0.218	0.106	0.154	−2.054	0.041
	PBAW	0.025	0.094	0.018	0.261	0.794
	CSC	−0.006	0.084	−0.004	−0.068	0.946
	NBUD	0.353	0.108	0.239	3.274	0.001
	NTCT	0.107	0.107	0.073	0.999	0.319

GAD: generalised anxiety; HIV: HIV stigma; Disc: discrimination; SAbuse: substance abuse; Lone: loneliness; SelfE: self esteem; LOCC: lack of cognitive confidence; PBAW: positive beliefs about worry; CSC: cognitive self consciousness; NBUD: negative beliefs about uncontrollability and danger; NTCT: need to control thoughts.

In the final equation, five cross sectional predictors made a unique and statistically significant contribution to GAD. Negative Beliefs about Uncontrollability and Danger (β = 0.24, *p* < 0.001) emerged as the strongest independent cross-sectional predictor of GAD, closely followed by discrimination (DISC-12; β = 0.23, *p* < 0.001). The only other metacognitive belief that emerged as a significant predictor of GAD was ‘Lack of Cognitive Confidence’ (β = 0.14, *p* < 0.05). Our other hypothesised variable ‘Positive Beliefs about Worry’ did not emerge as a significant predictor. The three other significant predictors of GAD were substance abuse (β = 0.21, *p* < 0.001), HIV stigma, (β = 0.18, *p* < 0.05) and loneliness (β = 0.12, *p* < 0.001).

To further explore the role of metacognitive beliefs in GAD within this population, we wanted to see the effect these beliefs had on HIV stigma and whether they increased symptoms of GAD. To explore this hypothesis a moderation model was tested with a moderator of the effect of X (HIV Stigma) on Y (GAD) by M (negative beliefs about uncontrollability and danger: NBUD). The moderator effect was highly significant B = 0.0078, 95% CI [0.0048, 0.0109], t = 4.357, *p* < 0.001.

Exploration of the conditional effect of *X* on *Y* at values of the moderator, revealed the following:

1. When NBUD is low there is a significant positive relationship between HIV stigma and GAD, *B* = 0.05, 95% CI [0.02, 0.07], *t* = 4.18, *p* < 0.001.
2. At the mean value of NBUD there is a strong significant positive relationship between HIV stigma and GAD, *B* = 0.09, 95% CI [0.07, 0.11], *t* = 8.28, *p* < 0.001.
3. When NBUD is high there is a stronger significant positive relationship between HIV stigma and GAD, *B* = 0.12, 95% CI [0.09, 0.15], *t* = 9.03, *p* < 0.001.

**Figure 1.**
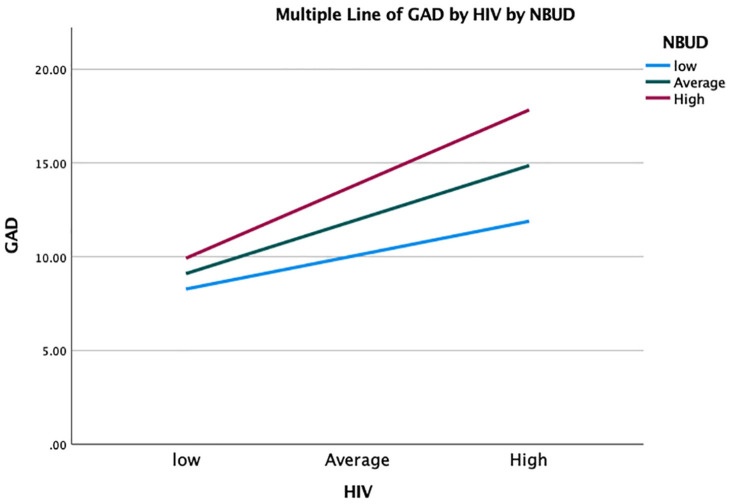
Simple slopes analysis. NBUD: negative beliefs about uncontrollability and danger; HIV: HIV stigma.

The simple slopes analysis (Figure 1) depicts these interaction effects. When NBUD total increases, the relationship between HIV stigma and GAD gets stronger and stays significant. In essence, this moderation means that type 1 worry that is, HIV stigma, contributes to GAD severity, as metacognitive beliefs increase.

## Discussion

The mental health of MSM with HIV in Lagos is of particular concern due to severe stigma, discrimination and legal restrictions against homosexuality in Nigeria (Dahlui et al., 2015). These social issues contribute to elevated rates of anxiety, depression and substance use in this population (Odimegwu et al., 2017). Identifying problematic psychological mechanisms that amplify these social factors are important to reduce levels of anxiety and help in the development of targeted mental health interventions for this group.

The aim of the study therefore was to identify whether metacognitive beliefs emerged as a significant predictor of GAD in a population of MSM with HIV in Lagos Nigeria whilst controlling for psycho-social factors, such as stigma, discrimination, substance abuse, loneliness and self-esteem. Furthermore, it tested the prediction that the effect of HIV stigma on GAD would be moderated by metacognition, in particular negative beliefs about the uncontrollability and danger of worry.

In relation to our first hypothesis, all five metacognitive beliefs were significantly correlated with GAD and supported our hypotheses that negative beliefs about uncontrollability (*r* = 0.60, *p* < 0.001) and positive metacognitive beliefs (*r* = 0.56, *p* < 0.001) would have the strongest association with GAD. This is in line with previous research that indicates a strong association between both positive and negative metacognitive beliefs and GAD (Sun et al., 2017; Thielsch et al., 2015; Wells, 2005). All other variables also had a significant positive association with GAD, with HIV stigma (*r* = 0.64, *p* < 0.001) and discrimination (*r* = 0.61, *p* < 0.001) emerging as the strongest correlations. These findings are consistent with previous research that indicate that self-stigma is associated with symptoms of GAD in MSM living with HIV (Hu et al., 2022), and that discrimination is associated with increased anxiety, and HIV risk behaviours (Rogers et al., 2018). The real-life implications of hypothesis 1 includes the recognition that MSM living with HIV with high levels of uncontrollability beliefs may struggle to manage their anxiety effectively, leading to heightened distress.

Our second hypothesis was partially supported where beliefs about uncontrollability and danger emerged as a significant predictor of GAD, although positive beliefs about worry did not. In this study we also controlled for all other psychosocial factors associated with GAD in MSM living with HIV, including stigma, discrimination, self-esteem, loneliness and substance abuse. All these variables, except self-esteem, where independent significant predictors of GAD, with both discrimination and negative beliefs about uncontrollability and danger being the strongest. Previous research indicates that beliefs about uncontrollability and danger consistently emerge as a significant predictor of both post situation worry (Fergus and Wheless, 2018) and GAD itself (Wells, 2005). In a comparison between individuals with or without GAD, those with GAD tended to have significantly higher negative metacognitive beliefs both cross sectionally (McEvoy et al., 2017) and longitudinally (Mennin et al., 2008). The real-life findings in our study are significant because they highlight not only that societal stigma and discrimination contribute directly to mental health challenges, but metacognitive beliefs may also play an important role in the development and maintenance of GAD in MSM living with HIV in Nigeria.

Beliefs about uncontrollability and danger may be problematic in this group as it can maintain stigma and discrimination based worry, as previous studies indicate these particular negative metacognitive beliefs lead to more content based worrying (Thielsch et al., 2015). For example, worry about being stigmatised or discriminated against could be more ubiquitous in this group than the actual experience of stigma and discrimination. In essence, MSM living with HIV may not necessarily need to be constantly experiencing discrimination and stigma in order to experience high levels of worry and anxiety (Deacon, 2006; Herek, 2002; Strodl et al., 2015). Also, if individuals believe that worrying is uncontrollable, they will be less inclined to try and stop, potentially leading to meta-worry that is, worry about worry (Wells and Matthews, 1994). So not only does HIV stigma and worry about stigma increase levels of anxiety, believing that you cannot control worry adds another level of anxiety (Ryum et al., 2017). According to the metacognitive model, both worry and meta-worry are central factors in individuals with GAD, and negative metacognitive beliefs contribute to both these processes (Nordahl et al., 2022).

Additionally, the belief that worrying is uncontrollable can contribute to increased predictions and responses to anxiety-related sense data (Wells et al., 2010). For MSM living with HIV, constantly monitoring cognition, scanning internally and externally for signs of discrimination and stigma and engaging in excessive worry as an attempt to maintain a sense of control, can often backfire (Wells and Cartwright-Hatton, 2004). Such hyper-vigilance and maladaptive coping responses can lead to an increase in anxiety and further reinforcement that worry is indeed uncontrollable (Sica et al., 2007). Furthermore, believing worry is uncontrollable can lead to maladaptive control behaviours such as substance abuse, which is consistent with this study and previous research where people living with HIV who have GAD symptoms tend to have elevated substance abuse problems (Mannes et al., 2021).

Contrary to our predictions, positive beliefs about worry did not emerge as a significant predictor of GAD in this group. This is at odds with the metacognitive model and other theoretical models which posit that positive metacognitive beliefs are integral to the development and maintenance of GAD, for example, intolerance of uncertainty models (e.g. Carleton et al., 2012). According to the metacognitive model, negative beliefs about uncontrollability and danger are always present in GAD, however positive beliefs about worry may not be. Wells (2005) states that positive beliefs about worry are experienced by everyone and may not be pathological or sufficient to lead to GAD.

Finally, we tested for a hypothesised moderator effect of metacognition, specifically negative beliefs about the uncontrollability and danger of worry. The result of the analysis showed that the effect of HIV stigma (a marker of content-based worry, i.e. type 1 worry) on GAD was explained by the interaction with metacognitive beliefs. According to this model, HIV stigma and HIV stigma-based worries are more likely to increase the symptoms of GAD because of their relationship with metacognition. Importantly, the more metacognitive beliefs increase the more stigmatised and anxious the person may feel. These findings are consistent with previous studies that have shown negative beliefs about uncontrollability and danger moderated the relationship between perceived stress and anxiety (Ramos-Cejudo and Salguero, 2017) and health worry and health anxiety (Bailey and Wells, 2015). The findings are also like another study which has shown that negative metacognitive beliefs about uncontrollability and danger mediate the relationship between HIV stigma and depressive and anxious symptoms (Strodl et al., 2015). In our study however, we explored moderation as we wanted to see whether the relationship between HIV stigma and GAD becomes stronger in the presence of metacognitive beliefs, rather than whether HIV stigma influences or is linked to metacognitive beliefs.

If these findings of our study are replicated, particularly prospectively, this may have some implications for treating this patient group. In a country like Nigeria which has draconian laws regarding homosexuality, low tolerance towards HIV (Dahlui et al., 2015), and supernatural perceptions of mental health causes (Labinjo et al., 2020), changing attitudes and reducing stigma may be a long evolving process (Odimegwu et al., 2017). Although some therapeutic interventions have shown preliminary success in reducing stigma and promoting HIV wellness/mental health of sexual and gender minorities, they have mainly focused on external factors rather than internal factors (Pulerwitz et al., 2024). Providing real life therapeutic interventions that also enhances controllability of worry and reduces anxiety may be beneficial for this patient group and enable some psychological relief without relying upon the slow progress of societal change. One therapy in particular which specifically targets problematic worry and negative metacognitive beliefs, and which may be helpful, is Metacognitive Therapy (MCT). MCT is currently the most effective treatment for GAD, showing better results when compared to CBT (Nordahl et al., 2018), intolerance of uncertainty therapy (van der Heiden et al., 2012), applied relaxation (Wells, 2011) and with effects lasting up to 9 years (Solem et al., 2021). Although available across Europe, this therapy is not widely available in any African nations including Nigeria, therefore training in this approach for therapists and further clinical research may well be advantageous for MSM living with HIV experiencing GAD.

## Limitations and future research

The study is not without limitations. The nature of the study is cross sectional and therefore no causal conclusions can be drawn. Future work should include a more robust prospective design. We also did not include a measure of meta-worry in our study which is an important component in the general metacognitive model of GAD and future studies should include this in any model testing. The study did not deeply explore how other intersecting factors, such as socioeconomic status, education or co-morbid conditions, might influence the relationship between metacognition, stigma and GAD. Future work should include other important intersecting factors. While established measures were used, their applicability and cultural validity in Nigerian populations, for example, the MCQ-30, may require further validation to ensure they accurately capture the constructs under investigation. A further limitation of this study is the lack of direct community involvement in the research process. Future research should incorporate participatory methods to strengthen the impact and applicability of the findings (Austin-Keiller et al., 2023).

Potential future research could evaluate the effectiveness of Metacognitive Therapy (MCT) in reducing GAD symptoms among MSM living with HIV in Nigeria. Randomised controlled trials (RCTs) could provide robust evidence for the applicability of this intervention in this population.
